# Ethnicity and race in access and usage of health and social care: results from a scoping review

**DOI:** 10.1093/geroni/igab046.1657

**Published:** 2021-12-17

**Authors:** Sandra Torres

**Affiliations:** Department of Sociology, Uppsala, Uppsala Lan, Sweden

## Abstract

Scholarship on ethnicity and old age is at a crossroad now that increased diversity is a given in older populations. The same holds true for the study of the role that ethnicity and race play in access and usage of health and social care in old age. This presentation relies on a scoping review of scholarship published between 1998 and 2020 that brings attention to the ways in which ethnicity & race – as grounds for stratification and disadvantage - are made sense of in this scholarship. The presentation will describe the topics that the review divulged, whether racism has been acknowledged in this scholarship so far, and how this has been the case. In doing so, this presentation will argue that if we are to address the inequalities that older ethnic minorities face we need not only a diversity-astute research agenda but also an injustice-aware one.

